# Bioinformatic gene analysis for potential biomarkers and therapeutic targets of atrial fibrillation-related stroke

**DOI:** 10.1186/s12967-019-1790-x

**Published:** 2019-02-13

**Authors:** Rongjun Zou, Dingwen Zhang, Lei Lv, Wanting Shi, Zijiao Song, Bin Yi, Bingjia Lai, Qian Chen, Songran Yang, Ping Hua

**Affiliations:** 1 Department of Cardio-Vascular Surgery, Sun Yat-sen Memorial Hospital, Sun Yat-sen University, Guangzhou, 510120 China; 2 Department of Gastroenterology, Fifth Affiliated Hospital, Sun Yat-sen University, Zhuhai, 519000 China; 3 Department of Endocrinology, Sun Yat-sen Memorial Hospital, Sun Yat-sen University, Guangzhou, 510120 China; 4 Department of Radiology, Sun Yat-sen Memorial Hospital, Sun Yat-sen University, Guangzhou, 510120 China; 5 The Second Department of Orthopedics, Affiliated Hospital of Beihua University, Jilin, 132011 China; 6 The Biobank of Sun Yat-sen Memorial Hospital, Sun Yat-sen University, Guangzhou, 510120 China; 7 Guangdong Province Key Laboratory of Brain Function and Disease, Zhongshan School of Medicine, Sun Yat-sen University, Guangzhou, 510080 China

**Keywords:** Gene analysis, Biomarkers, Atrial fibrillation-related stroke

## Abstract

**Background:**

Atrial fibrillation (AF) is one of the most prevalent sustained arrhythmias, however, epidemiological data may understate its actual prevalence. Meanwhile, AF is considered to be a major cause of ischemic strokes due to irregular heart-rhythm, coexisting chronic vascular inflammation, and renal insufficiency, and blood stasis. We studied co-expressed genes to understand relationships between atrial fibrillation (AF) and stroke and reveal potential biomarkers and therapeutic targets of AF-related stroke.

**Methods:**

AF-and stroke-related differentially expressed genes (DEGs) were identified via bioinformatic analysis Gene Expression Omnibus (GEO) datasets GSE79768 and GSE58294, respectively. Subsequently, extensive target prediction and network analyses methods were used to assess protein–protein interaction (PPI) networks, Gene Ontology (GO) terms and pathway enrichment for DEGs, and co-expressed DEGs coupled with corresponding predicted miRNAs involved in AF and stroke were assessed as well.

**Results:**

We identified 489, 265, 518, and 592 DEGs in left atrial specimens and cardioembolic stroke blood samples at < 3, 5, and 24 h, respectively. *LRRK2*, *CALM1*, *CXCR4*, *TLR4*, *CTNNB1*, and *CXCR2* may be implicated in AF and the hub-genes of *CD19*, *FGF9*, *SOX9*, *GNGT1*, and *NOG* may be associated with stroke. Finally, co-expressed DEGs of *ZNF566*, *PDZK1IP1*, *ZFHX3*, and *PITX2* coupled with corresponding predicted miRNAs, especially miR-27a-3p, miR-27b-3p, and miR-494-3p may be significantly associated with AF-related stroke.

**Conclusion:**

AF and stroke are related and *ZNF566*, *PDZK1IP1*, *ZFHX3*, and *PITX2* genes are significantly associated with novel biomarkers involved in AF-related stroke.

**Electronic supplementary material:**

The online version of this article (10.1186/s12967-019-1790-x) contains supplementary material, which is available to authorized users.

## Background

Atrial fibrillation (AF) is one of the most prevalent sustained arrhythmias, having an age-adjusted hospitalization incidence of 1–4% of the general population and an prevalence rising of > 13% for those older than 80-years-of-age [1, 2]. However, epidemiological data may understate its actual prevalence, because 40% of patients are asymptomatic and remain undiagnosed with subclinical AF [3]. There is also evidence that patients with AF have significantly increased cardiovascular-related morbidity, given its association with atrial and ventricular mechanical or electrical failure, structural and hemodynamic alterations, and thromboembolic events [3].

Stroke is the leading cause of disability and death and has an estimated incidence of 3.73 (95% CI 3.51–3.96) per 1000 person-years among black- and white- adults in an atherosclerosis risk in communities (ARIC) cohort [4]. Furthermore, global increases in stroke prevalence plus stroke-related disability and mortality associated with aging will increase [5, 6]. Thus, we may not now know the actual true burden of stroke due to limits in brain imaging identification in < 10 mm small hypointense areas and silent infarctions for 28% of those patients older than 65-years-of-age [7]. AF is commonly classified as paroxysmal, persistent or permanent, or new onset arrhythmia basing on the present continuous time, which mainly included that paroxysmal AF was self-terminates within 7 days, while persistent AF was lasts longer than 7 days or needs cardioversion, and usually has lasted for 3 months [8]. As we all kwon, AF is considering to be a major cause of ischemic strokes due to irregular heart-rhythm, coexisting chronic vascular inflammation, and renal insufficiency, and blood stasis. According to Rivaroxaban Once Daily Oral Direct Factor Xa Inhibition Compared With Vitamin K Antagonism for Prevention of Stroke and Embolism Trial in Atrial Fibrillation (ROCKET-AF) trial study, Steinberg et al. [9] suggested that the paroxysmal AF patients carrying a lower adjusted rate of stroke or systemic embolism (adjusted HR: 0.78, 95% CI 0.61–0.99, P = 0.045), all-cause mortality (adjusted HR: 0.79, 95% CI 0.67–0.94, P = 0.006), and the composite of stroke or systemic embolism or death (adjusted HR: 0.82, 95% CI 0.71–0.94, P = 0.005) than persistent AF patients after adjusted efficacy and safety outcomes. According to the Oxford vascular study (OXVASC), nearly 43.9% of ischemic strokes were associated with AF among patients 80 years-of-age or older who had a threefold increase in AF in the past 3 decades [10]. However, this assumption has been challenged by the atrial fibrillation reduction atrial pacing trial (ASSERT) which identified a temporal association between subclinical AF and stroke risk among patients with implantable pacemakers and defibrillators. They reported that only 8% and 16% of patients had an association between pre-detected and post-detected AF within months of stroke or systemic embolism, respectively [11]. Of note, AF is often intermittent and asymptomatic, and presents as an electromechanical disassociation of atrial fibrillation. Clinically, current stroke risk scores and traditional diagnosis with an electrocardiogram are practical, while the limitation of predict stroke risk accurately in individual AF patients was significantly identified, especially in persistent AF which carrying a higher risk of stroke or systemic embolism and all-cause mortality [12]. In this study, we identified co-expressed differentially expressed genes (co-DEGs) of persistent AF and stroke and elucidated molecular mechanisms and pathology of AF-related DEGs (AF-DEGs) and stroke-related DEGs (stroke-DEGs). Finally, we provide a bioinformatic analysis of DEGs and predicted microRNAs (miRNAs) for AF patients prone to stroke.

## Methods

### Materials and methods

GSE79768 and GSE58294 datasets were downloaded from GEO (http://www.ncbi.nlm.nih.gov/geo/) [13] and expression profiling arrays were generated using GPL570 (HG‑U133_Plus_2) Affymetrix Human Genome U133 Plus 2.0 Array (Affymetrix, Santa Clara, CA). Additionally, the GSE79768 dataset, including 26 specimens with paired left atrial (LA) and right atrial (RA) tissue obtained from 13 patients was used to identify differential LA-to-RA gene expression and molecular mechanisms for patients with persistent AF or sinus rhythm (SR) abnormalities and we describe potential mechanisms of AF-related remodeling in the LA and the relationship between LA arrhythmogenesis and thrombogenesis. In this study, persistent AF patients has lasts continuously for > 6 months, while the SR patients had no evidence of AF clinically and any anti-arrhythmic drug history. Blood samples of GSE58294 were collected from cardioembolic stroke (N = 69) and control patents (N = 23) at < 3, 5, and 24 h.

### Data processing

R packages of “affy”, “affyPLM”, and “limma” (http://www.bioconductor.org/packages/release/bioc/html/affy.html), provided by a bioconductor project [14], were applied to assess GSE79768 and GSE58294 RAW datasets. We used background correction, quantile normalization, probe summarization and log2‑transformation, to create a robust multi-array average (RMA), a log-transformed perfect match, and a mismatch probe (PM and MM) methods. The Benjamini‑Hochberg method was used to adjust original p-values, and the false discovery rate (FDR) procedure was used to calculate fold-changes (FC). Genes expression values of the|log2 FC| > 1and adjusted *p *< 0.05 were used for filtering AF-DEGs. However, the |log2 FC| > 1.5 and adjusted *p *< 0.05 were used to identify stroke-DEGs, given that blood sample specificity pointed to many genes. Additionally, we calculated and made Venn diagrams for co-DEGs for AF- and stroke-DEGs.

Finally, we applied online prediction tools utilizing microRNA Data Integration Portal (mirDIP) (http://ophid.utoronto.ca/mirDIP) [15], miRDB (http://mirdb.org/) [16], TargetScan (v7.1; http://www.targetscan.org/vert_71/) [17], and DIANA Tools (http://diana.imis.athena-innovation.gr/DianaTools/) [18], to predict potential microRNA targeting. Subsequently, we used the mirDIP, miRDB, TargetScan, and Diana Tools software to predict which of the selected miRNAs could target co-DEGs. We determined 5 top candidate miRNAs based on higher predicted scores for ≥ 3 prediction tools for each co-DEG.

### Identification of protein–protein interaction (PPI) networks of DEGs

PPI networks of AF- and stroke-DEGs were analyzed using the search tool for the retrieval of interacting genes (STRING database, V10.5; http://string-db.org/) that predicted protein functional associations and protein–protein interactions. Subsequently, Cytoscape software (V3.5.1; http://cytoscape.org/) was applied to visualize and analyze biological networks and node degrees, after downloading analytic results of the STRING database with a confidence score > 0.4 [19].

### Functional enrichment analysis

Gene Ontology (GO) and Kyoto Encyclopedia of Genes and Genomes (KEGG) pathway enrichment analyses of AF- and stroke-DEGs were carried out using the database for annotation, visualization and integrated discovery bioinformatics resources (DAVID Gene Functional Classification Tool, http://david.abcc.ncifcrf.gov/) [20], and REACTOME databases (v62; http://www.reactome.org) [21]. GO terms and KEGG maps of biological functions associated with a *p *< 0.05 was considered to be significantly enriched. In addition, we presented different biofunctions of AF- and stroke-DEGs in biological processes, molecular functions, and cellular components from DAVID and REACTOME databases, respectively.

Subsequently, the AmiGO database (v2.0; http://amigo.geneontology.org/amigo/) was used to analyze the GO consortium for selected co-DEGs to verify the accuracy and annotate biofunctions of identified co-DEGs [22]. Using microRNA target prediction, online tools from Diana-miRPath (v3.0; http://www.microrna.gr/miRPathv3) [18] were applied to evaluate interactions between miRNA previously identified using prediction tools and co-DEGs involved in AF and stroke.

### Identification of co-DEGs associated with nervous or cardiovascular diseases

The comparative toxicogenomics database (http://ctdbase.org/) was used to find integrated chemical-gene, chemical-disease, and gene-disease interactions to generate expanded networks and predict novel associations [23]. We used these data to analyze relationships between gene products and nervous or cardiovascular diseases. Here, relationships between co-DEGs and diseases and association or an implied association were identified.

## Results

### Identification of DEGs

**Fig. 1:**
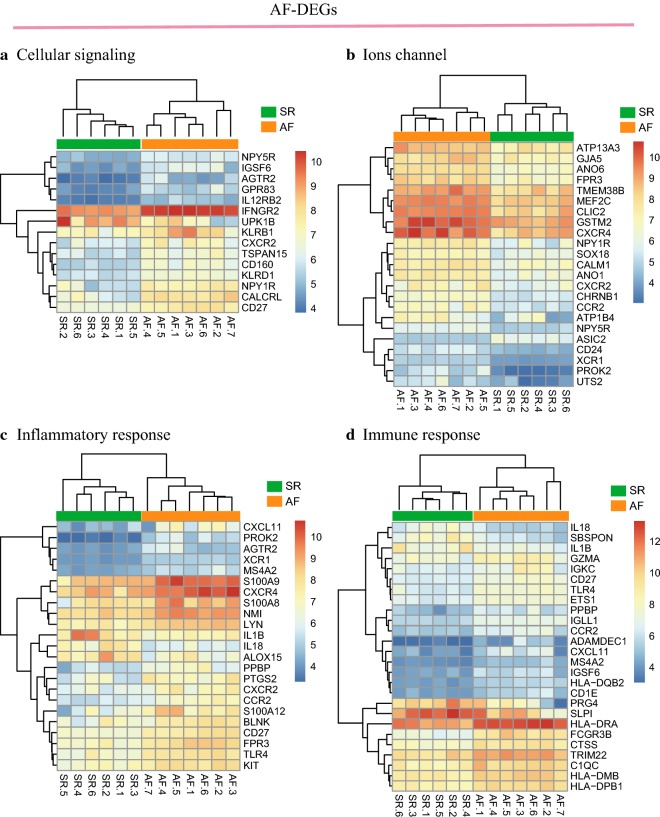
Hierarchical clustering analysis of AF-related differentially expressed genes: **a**–**d** results of hierarchical clustering analysis for DEGs expression in relation to cellular signaling, ion channel, inflammatory and immune responses. Red, greater expression. Blue, less expression

**Fig. 2:**
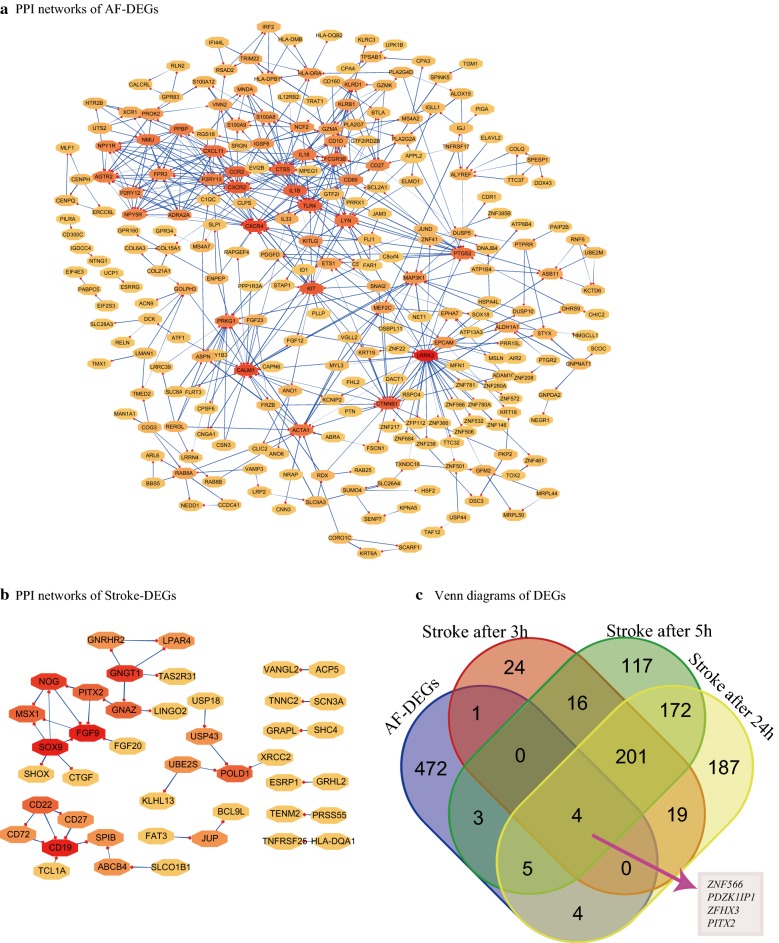
Hierarchical clustering analysis of stroke-related DEGs: **a**–**c** results of hierarchical clustering analysis for DEG expression in relation to energy metabolism, ion channel, inflammatory response, and neuronal regulation. Red, greater expression. Blue, less expression. **a** PPI network of AF-related DEGs; **b** PPI network of stroke-related DEGs. Red, greater degree. yellow, lesser degree; **c** Venn diagrams of DEGs

We identified 54,674 probes corresponding to 20,484 genes in GSE79768 and GSE58294 datasets and AF- and stroke-DEGs were confirmed. We found 489 DEGs in LA specimens of AF patients compared with SR patients, including 428 down-regulated genes and 61 up-regulated genes. However, total of 265, 518, and 592 DEGs were identified following the time points of less than 3, 5, and 24 h after stroke, respectively. Here, we defined 210 co expressed DEGs in the three time points mentioned above as the stroke-DEGs. Heatmaps of AF-DEGs in relation to inflammatory and immune response, ion channels, and cell signaling were conducted for genes expression and these data appear in Fig. 1 and Additional file 1: S1. Simultaneously, Fig. 2 and Additional file 2: S2 has shown the genes expression value in relation to inflammatory response, energy metabolism, ions channel and transportation, and neuronal regulation above the stroke-DEGs.

### Functional enrichment in Co-DEGs

**Fig. 3:**
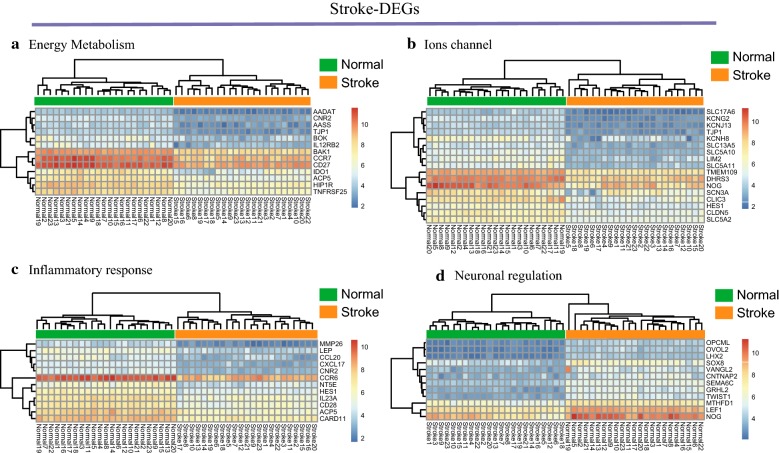
PPI network and Venn diagrams: (1) PPI networks from **a** and **b** constructed using STRING database for DEGs (threshold > 0.4). (2) Venn diagrams of **c** of DEGs related to AF and < 3, 5, and 24 h after stroke, respectively. Co-expressed genes, including ZNF566, PDZK1IP1, ZFHX3, and PITX2, are identified

Figure 3c illustrates expressed AF- and stroke-DEGs and co expressed genes. Interestingly, four co expressed DEGs, including zinc finger protein 566 (*ZNF566*), PDZK1 interacting protein 1(*PDZK1IP1*), zinc finger homeobox 3 (*ZFHX3*), paired-like homeodomain 2 *(PITX2*), were observed. The AmiGO database was used to confirm GO term enrichment related to biological processes, molecular functions, and cellular components and Co-DEGs were associated with various processes as indicated in Table 1.

**Table 1:** The Gene Ontology (GO) terms enrichment for the co-expressed genes of the AF-related stroke

Gene/product	GO class (direct)	Evidence	Evidence with	Reference
*PDZK1IP1*	Integral component of membrane	IEA	UniProtKB-KW:KW-0812	GO_REF:0000037
	Extracellular exosome	IDA		PMID:19056867
*ZNF566*	DNA binding	IEA	UniProtKB-KW:KW-0238	GO_REF:0000037
	Nucleus	IEA	UniProtKB-SubCell:SL-0191	GO_REF:0000039
	Transcription, DNA-templated	IEA	UniProtKB-KW:KW-0804	GO_REF:0000037
	Metal ion binding	IEA	UniProtKB-KW:KW-0479	GO_REF:0000037
*ZFHX3*	Negative regulation of transcription from RNA polymerase II promoter	IGI	UniProtKB:P01104	PMID:10318867
	RNA polymerase II proximal promoter sequence-specific DNA binding	IDA		PMID:7507206
	core promoter sequence-specific DNA binding	ISS	UniProtKB:Q61329	GO_REF:0000024
	Transcriptional repressor activity, RNA polymerase II proximal promoter sequence-specific DNA binding	IC	GO:0000122/GO:0000978	GO_REF:0000036
	Protein binding	IPI	UniProtKB:Q13761	PMID:20599712
	Nucleus	TAS		PMID:1719379
*PITX2*	Transcription regulatory region sequence-specific DNA binding	IDA		PMID:9685346
	Transcriptional activator activity, RNA polymerase II proximal promoter sequence-specific DNA binding	IEA	UniProtKB:Q9R0W1	GO_REF:0000107
	RNA polymerase II transcription coactivator activity	IDA		PMID:9685346
	Branching involved in blood vessel morphogenesis	IEA	UniProtKB:P97474	GO_REF:0000107
	Vasculogenesis	IEA	UniProtKB:P97474	GO_REF:0000107
	In utero embryonic development	IEA	UniProtKB:P97474	GO_REF:0000107
	Neuron migration	IEA	UniProtKB:P97474	GO_REF:0000107
	Extraocular skeletal muscle development	IEA	UniProtKB:P97474	GO_REF:0000107

*ISS* sequence similarity evidence used in manual assertion, *IGI* genetic interaction evidence used in manual assertion, *IDA* direct assay evidence used in manual assertion, *TAS* traceable author statement used in manual assertion, *IEA* evidence used in automatic assertion, *IPI* physical interaction evidence used in manual assertion

### PPI network analysis and functional GO terms and pathway enrichment analyses

We identified 256 and 43 nodes from PPI network of AF- and stroke-DEGs, respectively and these data appear in Fig. 3. Here, the hub nodes, including leucine-rich repeat kinase 2 (*LRRK2*; degree = 38), calmodulin 1 (*CALM1*; degree = 25), chemokine (C-X-C motif) receptor 4 (*CXCR4*; degree = 25), toll-like receptor 4 (*TLR4*; degree = 21), catenin (cadherin-associated protein), beta 1(*CTNNB1*; degree = 21), and chemokine (C-X-C motif) receptor 2 (*CXCR2*; degree = 21) are considering as hub-genes in related to AF maintaining. However, the hub-genes, involved in *CD19* (degree = 5), fibroblast growth factor 9 (*FGF9*; degree = 5), SRY (sex determining region Y)-box 9 (*SOX9*; degree = 5), guanine nucleotide binding protein (G protein), gamma transducing activity polypeptide 1(*GNGT1*; degree = 4), and noggin (*NOG*; degree = 4), are demonstrated in stroke-DEGs with a relative higher degree.

**Fig. 4:**
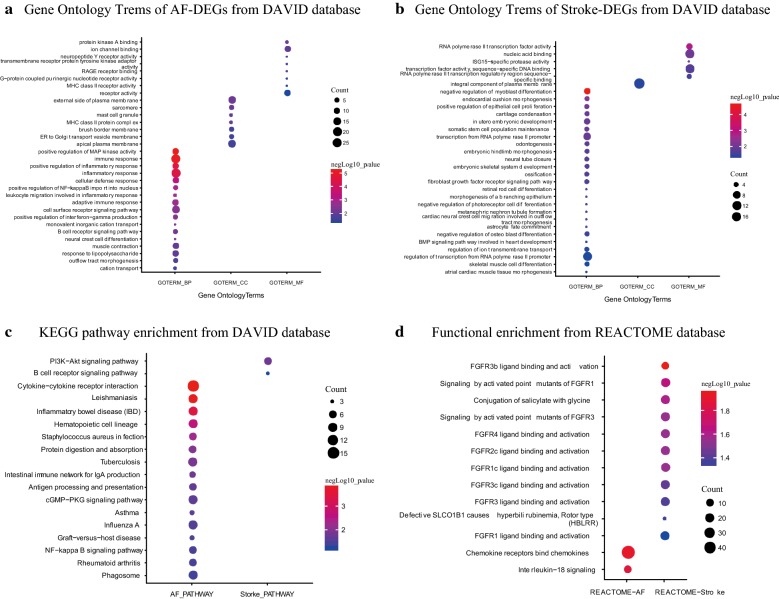
GO terms and KEGG pathway enrichment: **a**, **b** AF-and stroke-related GO term enrichment for DEGs, respectively. **c** KEGG pathway of AF- and stroke-related DEGs. **d** Functional and pathway enrichment of AF-and stroke-related DEGs from REACTOME database. Dot sizes represent counts of enriched DEGs, and dot colors represent negative Log10-p values

Using the DAVID database, the top 5 GO terms related biological processes among those genes were primarily associated with inflammatory response (Fold Enrichment: 4.08; *p* value: 1.11E−07), immune response (Fold Enrichment: 3.50; p-value: 2.49E−06), regulation of MAP kinase activity (Fold Enrichment: 9.53; p-value: 1.92E−05), and regulation of NF-kappa B activety (Fold Enrichment: 16.73; p-value: 1.95E−04). There is significant correlation in plasma membrane (Fold Enrichment: 1.60; p-value: 1.37E−06), extracellular region (Fold Enrichment: 1.75; p-value: 8.88E−04), and MHC class II protein complex (Fold Enrichment: 13.47; p-value: 0.003) in relation to cellular components. In addition, the terms related molecular functions were mainly involved in ion channel binding (Fold Enrichment: 5.06; p-value: 0.001), neuropeptide Y receptor activity (Fold Enrichment: 23.84; p-value: 0.007), and transmembrane receptor protein tyrosine kinase adaptor activity (Fold Enrichment: 21.46; p-value: 0.008). With respect to stroke-DEGs, the biological processes terms of regulation of myoblast differentiation (Fold Enrichment: 25.39; p-value: 4.89E−04), endocardial cushion morphogenesis (Fold Enrichment: 27.38; p-value: 0.005), positive regulation of epithelial cell proliferation (Fold Enrichment: 9.73; p-value: 0.008), and fibroblast growth factor receptor signaling pathway (Fold Enrichment: 7.12; p-value: 0.018) were significantly enriched. Similarly, the terms of RNA polymerase II transcription factor activity, sequence-specific DNA binding (Fold Enrichment: 5.15; p-value: 0.006), ISG15-specific protease activity (Fold Enrichment: 146.79; p-value: 0.013), and nucleic acid binding (Fold Enrichment: 2.09; p-value: 0.015) related molecular functions were primarily enriched (As shown in Fig. 4 and Additional file 3: S3).

**Fig. 5:**
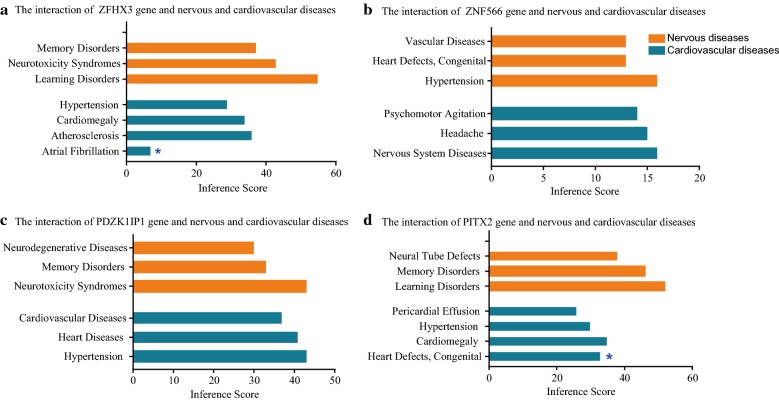
Relationship to nervous system and cardiovascular diseases related to co-expressed genes based on the CTD database. *Direct evidence of marker or mechanism in this disease

KEGG pathway analysis data appear in Fig. 4c. The results suggesting that the AF-DEGs were mainly enriched in pathways of cytokine–cytokine receptor interaction (p-value: 1.02E−04), cGMP-PKG signaling pathway (p-value: 0.025), antigen processing and presentation (p-value: 0.022), and NF-kappa B signaling pathway (p-value: 0.037). However, KEGG terms included PI3 K-Akt signaling pathway (p-value: 0.017) and B cell receptor signaling pathway (p-value: 0.045) were enriched in stroke-DEGs. (As shown in Fig. 4c and Additional file 4: S4).GO terms enrichment using the REACTOME database identified additional associations and these appear in Fig. 4d. The CTD database showed that Co-DEGs targeted several nervous system and cardiovascular diseases and these data appear in Fig. 5 and Additional file 5: S5.

### Identification of functional and pathway enrichment among predicted miRNAs and Co-DEGs

Prediction analysis using mirDIP, miRDB, TargetScan, and DIANA bioinformatic tools identified the top 5 selected miRNAs targeting each Co-DEG involved in AF-related stroke and these data appear in Table 2. These data enable us to understand how predicted miRNAs are related to AF-related stroke progress.

**Table 2:** The Gene Ontology (GO) terms and Kyoto Encyclopedia of Genes and Genomes (KEGG) pathways enrichment among predicted miRNAs and Co-DEGs

Genes	Predicted miRNAs	Category		P value
*PDZK1IP1*	hsa-miR-1296-5p	KEGG pathway	Hypertrophic cardiomyopathy (HCM)	0.047
	hsa-miR-27a-3p		NF-kappa B signaling pathway	0.022
	hsa-miR-27b-3p		Toll-like receptor signaling pathway	0.017
	hsa-miR-6895-5p	GO terms	Negative regulation of energy homeostasis	4E−04
	hsa-miR-4725-3p		Calcium ion-dependent exocytosis of neurotransmitter	0.001
			Neurotransmitter receptor activity	0.002
			Regulation of leukocyte migration	0.003
			Smooth muscle hyperplasia	0.011
			Immunoglobulin mediated immune response	0.034
*ZNF566*	hsa-miR-216b-5p	KEGG pathway	Valine, leucine and isoleucine biosynthesis	0.320
	hsa-miR-1277-5p		Cardiac muscle contraction	0.411
	hsa-miR-6783-5p	GO terms	Regulation of acute inflammatory response	0.007
	hsa-miR-369-3p		Commissural neuron differentiation in spinal cord	0.019
	hsa-miR-6778-3p		Cardiac vascular smooth muscle cell differentiation	0.020
			MHC protein binding	0.035
			ATP-activated inward rectifier potassium channel activity	0.035
			Mitochondrial translation	0.041
*PITX2*	hsa-miR-377-3p	KEGG pathway	TGF-beta signaling pathway	3.00E − 03
	hsa-miR-141-3p		Phosphatidylinositol signaling system	0.124
	hsa-miR-5692b	GO terms	Vascular smooth muscle cell differentiation	0.002
	hsa-miR-4789-5p		Cell proliferation involved in outflow tract morphogenesis	0.002
	hsa-miR-494-3p		Cardiac neural crest cell migration involved in outflow tract morphogenesis	0.002
			Pulmonary myocardium development	0.002
			Atrial cardiac muscle tissue morphogenesis	0.002
			Ventricular cardiac muscle cell development	0.003
			Cardiac muscle cell differentiation	0.006
			Neuron migration	0.014
			Neuron differentiation	0.014
*ZFHX3*	hsa-miR-494-3p	KEGG pathway	Regulating pluripotency of stem cells	3E − 07
	hsa-miR-758-3p	GO terms	Positive regulation of myoblast differentiation	0.023
	hsa-miR-27a-3p		Negative regulation of myoblast differentiation	0.023
	hsa-miR-27b-3p		Regulation of neuron differentiation	0.023
	hsa-miR-493-5p		Muscle organ development	0.041

## Discussion

Predicting AF is needed for stroke prevention but 30% of patients have no signs of AF despite months of continuous cardiac rhythm monitoring. Thus, cardiovascular malignant events may be correlated with irregular and infrequent cardiovascular incidents as well as limitations in electromechanical indices that should predict problems with atrial contractility [7, 11, 12]. Estimating markers and associations between atrial dysfunction and embolic stroke are thus of interest and may be novel therapeutic targets for primary care. The inflammatory and immune response, and ion channel and transportation are significantly associated with AF recurrence and maintenance, as well as the stroke occurrence. Several hub-genes involved directly or indirectly that regulate the nervous system were found among AF-DEGs. Visanji’s group compared resting electrocardiograms of *LRRK2*-associated Parkinson’s disease (PD) patients, nonmanifesting carriers, noncarriers, and idiopathic PD patients to investigate heart rate variability in *LRRK2*-associated PD [24]. There is evidence that *LRRK2* may act as a biofunctional mediator to correlate heart rate variability and PD [24]. In a molecular mechanistic study, the neural protective role for regulating mitochondrial complex I function and oxidative stress in ischemia/reperfusion was identified [25, 26]. According gene–gene interaction analysis, Timasheva’s group illustrated that the loci of *CXCR2* is significantly associated with stroke development in patients with hypertension [26]. In addition, *CXCR2* antagonism attenuated neurological deficits and infarct volumes via decreased cerebral neutrophil infiltration and peripheral neutrophilia in a hyperlipidemic ApoE^−/−^ mice stroke model [27]. *CALM1* is recognized as a major regulator of cardiac ion-current expression and calcium handling, and a key determinant of cardiac electrical function [28]. Also, specific risk alleles for *CALM1* were identified as being associated with increased risk of stroke in studies of coronary heart disease [29]. Thus, there may be a relationship between cardiovascular and nervous system disease and they may arise from loci mutations or gene variants.

Additionally, *PITX2*, of the pituitary homeobox (Pitx) family, has a critical role in organ morphogenesis and AF maintenance which is related to short stature homeobox 2 (*Shox2*) [30]. *Pitx2* is expressed in the LA and the pulmonary vein, which is considered a substrate and trigger for AF maintenance respectively. However, several experimental data indicate a trend that *PITX2* gene expression is silenced during aging in LA samples, suggesting genetic evidence for gene silencing for increased AF susceptibility [30, 31]. Then, miRNAs function analysis and a genomic approach showed that miR-17-92 and miR-106b-25 were associated with Pitx2 expression regulation and are implicated in human AF susceptibility [31]. To reveal relationships between genetic variants and the risk of ischemic stroke, Malik’s group studied *PITX2* and *ZFHX3* genes and found a significant association with cardioembolic stroke (CE) in a meta-analysis [31, 32]. Similarly, in a genome-wide association study using clinical samples from paroxysmal or persistent AF patients, *ZFHX3* was significantly associated with LA enlargement and persistent AF and subsequently with ablation outcomes [33]. Correspondingly, Choi’s group found a significant association between top susceptibility loci (chromosomes 4q25 [*PITX2]*, 16q22 [*ZFHX3*]) and AF recurrence after ablation in a Korean population, despite no top single nucleotide polymorphisms (SNPs) that predicted clinical recurrence after catheter ablation [34]. A regulatory role for *PDZK1IP1* (*MAP17*) in reactive oxygen species production has been confirmed and is considered as a marker for increased oxidative stress and may be a new therapeutic target [35]. and recent research suggests a potential role for ions channels regulation, linked to the Na^+^/H^+^ exchanger 3 and A-kinase anchor protein 2/protein kinase A pathway [36]. However, *ZNF566* plays a central role in heart regeneration and repair, and endocardial and epicardial epithelial to mesenchymal transitions [37, 38].

Research suggests potential beneficial effects of miRNA transformation therapy vectored by adenovirus, plasmid, and lentivirus for AF therapy [39]. We found that miR-27a-3p, miR-27b-3p, and miR-494-3p were co-DEGs and may be potential biomarkers of AF-related stroke. Interestingly, Vegter’s group compared heart failure-specific circulating miRNAs in 114 heart failure patients with/without different manifestations of atherosclerotic disease, and reported that miR-18a-5p, miR-27a-3p, miR-199a-3p, miR-223-3p and miR-652-3p abundance were associated with atherosclerosis and cardiovascular-related rehospitalizations [40]. Similarly, Marques and colleagues found that several miRNAs involved in let-7b-5p, let-7c-5p, let-7e-5p, miR-122-5p, and miR-21-5p, and absorbed miR-16-5p, miR-17-5p, miR-27a-3p, and miR-27b-3p are target pathways related to heart failure and considered to be potential biomarkers [41]. In contrast, expression of miR-27b-3p is significantly related to embryonic myogenesis and protein synthesis but miR-494-3p expression is associated with cerebral blood supply and functional recovery in a rat stroke model according to cerebral cortical miRNA profile changes [42, 43].

## Conclusion

The hub-genes of *LRRK2*, *CALM1*, *CXCR4*, *TLR4*, *CTNNB1*, *CXCR2*, *KIT*, and *IL1B* may be associated with AF recurrence and maintenance and *CD19*, *FGF9*, *SOX9*, *GNGT1*, and *NOG* may be associated with stroke. Additionally, co-DEGs of *ZNF566*, *PDZK1IP1*, *ZFHX3*, and *PITX2* link AF and stroke. Finally, the top 5 miRNAs for each co-DEGs may be potential biomarkers or therapeutic targets for AF-stroke, especially miR-27a-3p, miR-27b-3p, and miR-494-3p. Thus, there is an association between AF and stroke, and expression of *ZNF566*, *PDZK1IP1*, *ZFHX3*, and *PITX2* genes favor AF-related stroke.

## Limitation

Several limitations still detected in our study. First, this study is a microarray analysis that all the results based on gene expression value. However, owing to gene expression may be not directly equivalent to protein expression, the biomarkers of this study should consider as gene, not in protein. In application, assay of PCR and microarray chip may be better for accessing the risk of AF-related stroke. Second, validation should be carried out both in vitro, in vivo and clinical trials. However, as of now the techniques of in vivo or in vitro models for AF and stroke was immature. And the larger, prospective clinical studies may be better to validate our results to some extent.

## Additional files

**Additional file 1: S1.** Differentially expressed genes involved in AF samples.

**Additional file 2: S2.** Differentially expressed genes involved in stroke.

**Additional file 3: S3.** Gene Ontology (GO) terms enrichment analysis of AF- and stroke-related differentially expressed genes.

**Additional file 4: S4.** Kyoto Encyclopedia of Genes and Genomes (KEGG) pathway enrichment analysis among the AF- and stroke-related differentially expressed genes.

**Additional file 5: S5.** Relationship between diseases and co-expressed genes.

## Abbreviations

AF: atrial fibrillation
DEGs: differentially expressed genes
GEO: gene expression omnibus
PPI: protein–protein interaction
GO: Gene Ontology
ARIC: atherosclerosis risk in communities
OXVASC: the Oxford vascular study
ASSERT: atrial fibrillation reduction atrial pacing trial
co-DEGs: co-expressed differentially expressed genes
AF-DEGs: AF-related differentially expressed genes
stroke-DEGs: stroke-related differentially expressed genes
miRNA: microRNA
LA: left atrial
RA: right atrial
SR: sinus rhythm
RMA: robust multi-array average
PM: perfect match
MM: mismatch probe
FDR: false discovery rate
FC: fold-changes
mirDIP: microRNA Data Integration Portal
KEGG: Kyoto Encyclopedia of Genes and Genomes
PD: Parkinson’s disease
Pitx: pituitary homeobox
*Shox2*: short stature homeobox 2
CE: cardioembolic stroke
SNPs: single nucleotide polymorphisms
